# Endocrine Function in Aging

**DOI:** 10.1155/2012/872478

**Published:** 2012-12-30

**Authors:** Huan Cai, Alan S. Mcneilly, Louis M. Luttrell, Bronwen Martin

**Affiliations:** ^1^Metabolism Unit, National Institute on Aging, National Institutes of Health, 251 Bayview Boulevard, Baltimore, MD 21224, USA; ^2^MRC Centre for Reproductive Health, The Queen's Medical Research Institute, University of Edinburgh, 47 Little France Crescent, Edinburgh EH16 4TJ, UK; ^3^Division of Endocrinology, Diabetes & Medical Genetics, Department of Medicine, Medical University of South Carolina, Charleston, SC 29425, USA

The endocrine system in higher mammals represents one of the most complex and fundamental systems that regulates nearly all of an organism's biological functions. This system is composed of multiple organs, tissues, hormones, and receptor modalities. Its ability to regulate critical functions such as reproduction, development, metabolism, stress responses, blood pressure, wakefulness, and digestion places it as one of the most important regulators of life-long physiology. Therefore, at any one point in time the physiological status of the majority of organs in the body is a function of the activity of the whole endocrine system. However, while appreciating the role of the endocrine system in such “frozen” points in time is important, the temporal variation in endocrine function across one's lifespan is of crucial interest to researchers investigating age-related disorders. The importance of gerontological research is becoming more and more evident, given the ever-increasing proportion of aged people in Western countries. 

Aging is a natural process that involves a general decline in many physiological functions with time. Aging is generically associated with a reduced capacity to maintain homeostasis and effective repair mechanisms, resulting in loss of function, senescence, and eventually death. It is obvious that the functions of endocrine organs alter during the aging process, resulting in a higher prevalence of endocrine malfunction-related disorders in the elderly population. Enhanced knowledge and appreciation of endocrine functions in aging will likely lead to the development of successful pharmacological or lifestyle therapies to treat endocrine-related diseases in elderly patients. The discovery and development of novel endocrine-targeted remedies will hopefully result in an improvement of quality of life and also overall lifespan. Thus, endocrine functions in the aging context are important fields of intense clinical and scientific interest and form the focus of this special issue. 

The papers in this special issue are focused upon original research papers and review papers concerning several important molecular and tissue systems vital to the maintenance of the endocrine system in aging, that is, pancreatic function and type 2 diabetes mellitus (T2DM); testosterone deficiency and depression; metabolic and endocrine alterations in muscle dystrophies and sarcopenia; serum adipokines and osteocalcin in older patients with hip fracture; gerontological neuroendocrine axis organization and disruption; correlation of thyroid hormones and lipid profiles in elderly T2DM patients; minimally invasive approaches to parathyroid surgery in elderly patients; the proper interpretation of hormones and tumor marker measurements in the geriatric population.

As we have stated, aging is an important risk factor for metabolic disorders, including obesity, impaired glucose tolerance, and type 2 diabetes. Aging has long been associated in multiple animal species with the insulin/insulin-like growth factor-1 (IGF-1) signaling system. Z. Gong and R. H. Muzumdar summarize in their paper the current evidence on how aging affects pancreatic*β*-cell function, *β*-cell mass, insulin secretion, and insulin sensitivity. They also review the effects of aging on the relationship between insulin sensitivity and insulin secretion. Accelerated insulin resistance appears to be one of the strongest hallmarks of advanced physiological aging; therefore, a comprehensive understanding of all the defects that impair glucose homeostasis in the elderly will likely lead to the development of novel treatments that may substantially improve life quality and lifespan. 

Testosterone deficiency, or hypotestosteronemia, is a widely recognized hormonal alteration strongly associated with male aging. The review paper by M. Amore et al. comprehensively summarizes the current understanding of the correlation between depressive symptoms with a syndrome called partial androgen deficiency of the aging male (PADAM). This paper highlights the potential benefits of testosterone treatment upon mood and affective disorders. While supplementation with testosterone fails to show sound evidence of effectiveness in the treatment of depression, testosterone supplementation has proved to be effective, on some levels, for improving quality of life of aged patients with PADAM. 

In addition to the gerontological effects upon steroid hormone activity, aging also significantly affects thyroid function. These age-related effects, acting through the thyroid hormone system, greatly impact both lipid profiles and somatic metabolic parameters. The study conducted by F. Strollo et al. investigates the correlation between free thyroxine (FT_4_), free triiodothyronine (FT_3_) levels and total cholesterol (TC), and low-density lipoprotein cholesterol (LDL-C) levels in euthyroid elderly T2DM patients. They found that TC and LDL-C correlate negatively with FT_4_ and positively with FT_3_. When divided according to treatment by oral hypoglycemic agents (OHA) and insulin (IT), they, however, reacted differently with respect to investigated associations.

Primary hyperparathyroidism (pHPT) is one of the most common endocrine diseases in the elderly and the chance of developing pHPT increases with age. Elderly patients with pHPT are often not referred for surgery because of their associated comorbidities that may increase surgical risk.The study by C. Dobrinja et al. demonstrates that minimally invasive approaches to parathyroid surgery seem to be safe and curative in elderly patients, with few associated risks because of the combination of modern preoperative imaging, advances in surgical technique, and advances in anesthesia care.

One of the most important factors related to the maintenance of health and independence in the elderly is endocrine-mediated control of the musculo skeletal system. An inability to maintain independence as well as increased morbidity due to elevated fall episodes are both likely to severely impact the cost of widespread healthcare in aging Western societies. Therefore, we have included several sections that contend with the effects of aging upon the endocrine regulation of musculo-skeletal tissues. 

Common metabolic and endocrine alterations exist across a wide range of muscular dystrophies. The paper by O. del Rocío Cruz Guzmán et al. expertly reviews the current knowledge concerning the metabolic and endocrine alterations in diverse types of dystrophinopathies including childhood and adult dystrophies. K. Sakuma and A. Yamauchi also review the vital pieces of data concerning our current understating of the endocrine contribution to the age-related declines in muscle mass, muscle strength, and sarcopenia. These authors also investigated the current hormonal interventions designed to improve endocrine defects related to sarcopenia. Myostatin inhibition seems to be an intriguing strategy for attenuating sarcopenia as well as muscular dystrophy. The authors discussed how therapeutic supplementation with growth hormone, IGF-I, or estrogen had a minor sarcopenia-inhibiting effect, and that testosterone supplementation in large doses had several side effects, even though it significantly improved muscle defects. Ghrelin mimetics could also potentially be beneficial and reverse the dysfunctional catabolic state associated with sarcopenia in the elderly population. 

Low bone mass density, a classical age-related health issue and a known health concern for fair-skinned, thin, postmenopausal Caucasian women, is found to be common among individuals with developmental/intellectual disabilities. The review paper by J. Jasien et al. provides a comprehensive overview of bone health of adults with developmental/intellectual disabilities, their risk of fractures, and how this compares to the general aging population. The authors contend that gaining a greater understanding of how bone health affected in individuals with developmental/intellectual disabilities could lead to better customized treatments for these specific populations. 

The paper by A. Fisher et al. reveals the interactions between serum adipokines and osteocalcin in older patients with hip fracture. The authors found that serum osteocalcin concentration was inversely associated with resistin and positively with leptin, leptin/resistin ratio, and adiponectin/resistin ratio after adjustment for multiple potential confounders. Osteocalcin was found to be an independent predictor of serum leptin, resistin, leptin/resistin, and adiponectin/resistin ratios, which suggests bidirectional interactions (crosstalk) between leptin, resistin, and osteocalcin as a part of a complex homeostatic system regulating bone and energy metabolism.

The accuracy of analytical measurements of different biochemical parameters is of vital importance for the proper diagnosis and treatment monitoring of elderly patients. The paper by K. Sztefko et al. discusses important points to be considered in the interpretation of hormone and tumor marker measurements in the geriatric population using immunochemical methods, including general lack of immunoassay standardization, presence of cross-reacting substances in patients' samples, limitation of free hormone measurements due to abnormal analyte binding protein concentrations, assay interferences due to a patient's autoantibodies, heterophilic antibodies, and proper interpretation of very low- and very-high-sample analyte levels.

While the endocrine system is classically associated with the regulation of autonomic hormonal functions, many lines of recent evidence have demonstrated that cognitive central nervous function in the elderly is significantly affected during the aging process by endocrine control of somatic metabolism. Hence, both normal and pathophysiological aging, as well as neurodegenerative disorders, are all influenced by this “neurometabolic” interface. This functional connection between these two important systems (neuronal and endocrine) is primarily mediated through hormonal communication between the brain and the metabolic organs. The review paper by S. Siddiqui et al. discusses the physical structure and molecular components of this fundamental “neurometabolic” axis in aging. The authors then elaborate upon this by discussing how the connection of these two major functional domains is likely to be created by multifunctional “keystone” signaling factors, such as the epidermal growth factor receptor (EGFR). This paper draws together evidence to aid the appreciation of the truly multidimensional role of EGFR, at the systemic level, in neurometabolic processes and in the neurodegenerative trajectories seen in the aging process.

In another paper that discusses the functional interface between neuronal and endocrine systems during the aging process, A. M. Stranahan et al. investigate the effect of two well-characterized antiaging interventions (caloric restriction or exercise) upon hypothalamic function. The hypothalamus forms a vital bridge between higher neuronal activity and the status of the peripheral endocrine hormone system. Age-related changes in hypothalamic activity appear to be strongly connected to both endocrine and neuronal pathophysiological mechanisms. A. M. Stranahan et al. employ both caloric restriction and voluntary wheel running paradigms in diabetic and nondiabetic animals to investigate the contextual sensitivity of hypothalamic transcriptomic responses to these antiaging lifestyle strategies. The authors found that caloric restriction and physical exercise were associated with distinct hypothalamic transcriptional signatures that differed significantly between the host physiological contexts of the diabetic or nondiabetic mice.

In conclusion, our understanding of endocrine function in aging is making great strides. Several timely topics concerning endocrine function in aging were purposefully included in this special issue. However, it is clear that further efforts are needed to gain a greater appreciation of the mechanisms underlying endocrine alterations in aging, which will aid the development of more effective interventions for the treatment of endocrine defects during the aging process.

